# Relationship between impaired adipogenesis of retroperitoneal adipose tissue and hypertrophic obesity: role of endogenous glucocorticoid excess

**DOI:** 10.1111/jcmm.12308

**Published:** 2014-06-09

**Authors:** María G Zubiría, Juana Vidal-Bravo, Eduardo Spinedi, Andrés Giovambattista

**Affiliations:** Neuroendocrine Unit, IMBICE (CONICET La Plata-CICPBA)La Plata, Argentina

**Keywords:** MSG rat, visceral adiposity, SVF cells, pro-/anti-adipogenic signals, cell lipid, adipokines, ADX, HRT

## Abstract

Although the pro-adipogenic effect of glucocorticoid (GC) on adipose tissue (AT) precursor cell differentiation is openly accepted, the effect of chronically high peripheral levels of GC on AT mass expansion is not fully understood. In the present study, we aim to assess the *in vitro* adipogenic capacity of AT precursor cells isolated from retroperitoneal (RP) AT pads of the hypercorticosteronaemic, adult neonatally treated monosodium L-glutamate (MSG) male rat. To ascertain this issue, we explored the *in vitro* adipogenic process of stromal-vascular fraction (SVF) cells isolated from RPAT pads of 60-day-old MSG rats. The data recorded indicated that RPAT-SVF cells from hypercorticosteronaemic MSG rats, although displaying an enhanced proliferation capacity, differentiated slower than normal cells. This dysfunction was associated with a reduction in key parameters indicative of precursor cell commitment, differentiation capacity and the percentage of fully differentiated adipocytes, with a retarded maturation process. The distorted adipogenic capacity was highly conditioned by RPAT-SVF cells displaying a low committed population and both excessive and reduced expression of anti- (Pref-1 and Wnt-10b) and pro-adipogenic (mineralocorticoid receptor) signals respectively. Notably, the normalization of peripheral corticosterone levels in MSG rats, as a result of bilateral adrenalectomy combined with GC replacement therapy, fully prevented reduced RPAT precursor cell commitment and overall impaired adipogenesis. Our study strongly supports that the impaired adipogenic process observed in the adult hypertrophic obese MSG male rat is a GC-dependent mechanism, thus explaining the unhealthy RPAT expansion observed in human hypertrophic obese phenotypes, such as in the Cushing's syndrome.

## Introduction

Adipogenesis is a highly ordered temporal sequence of events occurring at the adipose tissue (AT) precursor cell level. AT committed cells – Zfp423 and peroxisome proliferator-activated receptor (PPAR)-γ2-positive cells [1] – undergo terminal differentiation, which includes: (a) activation of the pivotal pro-adipogenic cell structure, mineralocorticoid receptor (MR) [2], (b) reduction in the cell expression of anti-adipogenic signals [*e.g*. pre-adipocyte factor-1 (Pref-1) and wingless-type MMTV integration site family, member 10b (Wnt-10b)] [3] and (c) increase in the cell expression of pro-adipogenic factors [*e.g*. PPAR-γ2, CBEP/α] [4]. The adipogenic process can be reproduced *in vitro* mainly by stimulating MR [2] in AT stromal-vascular fraction (SVF)-committed cells with dexamethasone (DXM) and insulin [5].

It has been reported that rats treated with monosodium L-glutamate (MSG) at neonatal age develop hyperadiposity [6] and neuroendocrine dysfunctions [7]. It is true that the adult MSG rat shares several characteristics with the human phenotypes of hypertrophic obesity, namely that of the Cushing's syndrome. Among them are hyperleptinaemia [8], increased visceral AT (VAT) mass and cell size [9,10], and excessive production of glucocorticoid (GC) [11,12]. MSG treatment damages hypothalamic arcuate nucleus (ARC) neurons [7] in charge of energy homeostasis control. Consequently, the cross-talk between hypothalamo-pituitary-adrenal (HPA) axis and AT functions [11,12] becomes disrupted. In fact, an early development of enhanced adrenal GC production [10,11] increased leptinaemia [13]; thus, these rats develop adrenal leptin-resistance [11,13]. Thereafter, a worsening in the metabolism of the adult MSG rat is because of the development of hyperinsulinaemia [8,14] and reduced catecholamine production [15]. As a result, VAT adipocytes of MSG rats became hypertrophic, insulin resistant and over-produce both leptin [8] and lipids [16]. In turn, hyperlipidaemia [17] and ectopic lipid deposition [18] aggravate this phenotype.

We earlier reported that several metabolic-neuroendocrine dysfunctions of the MSG rat are dependent on enhanced GC production [8,11,14]. Because most of the obesity-associated metabolic disorders are dependent on VAT dysfunction, the aim was to explore in the adult MSG rat whether: (a) the endogenous GC-rich milieu could impact on the *ex vivo* adipogenic capacity of retroperitoneal AT (RPAT) SVF cells; and (b) the normalization of corticoadrenal hyperactivity could be crucial for further amelioration of unhealthy AT expansion.

## Materials and methods

### Animals

Male newborn Sprague–Dawley rats were injected i.p with either 4 mg/g BW MSG (Sigma Chemical CO., St. Louis, MO, USA; dissolved in sterile 0.9% NaCl) or 10% NaCl (litter-mate controls; CTR) on alternate days between 2 and 10 days of age [14]. Weaned rats (21 days of age) were individually caged and kept in a light (lights on between 7 a.m. and 7 p.m.)- and temperature (22°C)-controlled room; rat Purina chow (Ganave, Argentina) and water were available *ad libitum* until experimentation (age 60 days). MSG-injected animals were screened for effectiveness of treatment by: hypophagia, decreased hypothalamic NPY mRNA expression and macroscopic observation of degeneration of the optic nerves at the time of sacrifice [14]. In each experiment, CTR and MSG rats were members of the same litters; however, when accumulating experiments, each different experiment was performed with animals from different litters. Animals were killed by decapitation in non-fasting condition (8–9 a.m.), and trunk blood was collected into EDTA-coated tubes. Tubes were rapidly centrifuged (4°C; 2500 × g; 15 min.) and plasma samples kept frozen (−20°C) until metabolite measurements. We have chosen the RPAT pad for the reason that is a non-visceral fat pad, closely related for paracrine interaction with adrenal corticosteroids, and with a single vagal innervation. Our Institutional Animal Care Committee approved all experiments. Animal manipulation followed protocols for animal use, in agreement with NIH Guidelines for care and use of experimental animals.

### Experimental designs

#### Experiment 1

RPAT pads from CTR and MSG rats were aseptically dissected, weighed and placed in sterile Petri dishes containing 10 ml of sterile DMEM medium. Pads were then used in several experiments, as described below.

*Morphometric studies in RPAT pads*. Freshly dissected AT pads were immediately fixed in 4% paraformaldehyde (in 0.2 M phosphate buffer), at 4°C for a maximum of 3 days. Tissues were then washed with 0.01 M PBS and immersed in 70% ethanol for 24 hrs before being processed and embedded in paraffin. Sections of 4 μm were obtained at different levels of the blocks and stained with haematoxylin–eosin, then examined with a Jenamed 2 Carl Zeiss light microscope; quantitative morphometric analysis was performed with a RGB CCD Sony camera together with the OPTIMAS software (Bioscan Incorporated, Edmons, WA, USA; 40× objective). For each RPAT sample (*n* = 4/5 animals per group), systematic random sampling was used to select 10 fields for each section and a minimum of 100 cells per group were examined. Each field of cells in a reference area (RA: tissue area scanned where adipocytes were scored) was measured for an average of 10 micrographs taken from two different levels. These measurements were recorded and processed, and two parameters were then calculated: cell density (CD: number of adipocytes/RA) and cell size (CS; expressed in μm^2^) [8].

*RPAT-SVF cell isolation and proliferation*. Pads were processed as previously described [19]. Briefly, pads were minced and digested using 1 mg/ml collagenase solution in DMEM (37°C–1 hr). After centrifugation (280 × g, 15 min.), floating adipocytes were discarded and the SVF cell-pellet was collected, filtered (in a 50-μm mesh nylon cloth) and washed (×2) with DMEM. Isolated SVF cells were then seeded (density: 15,000 cells/well; 24-well plates) and cultured in DMEM supplemented with HEPES (20 nM), 10% (vol/vol) foetal bovine serum (FBS), 100 U/ml penicillin and 100 μg/ml streptomycin at 37°C in a 5% CO_2_-atmosphere. Cells were left in culture to proliferate up to 9 days (Proliferation day: Pd 9). Every 24 hrs, cells (4 wells per day) were washed (×1) with PBS buffer. Thereafter, a 0.25% (w/v) trypsin solution (dissolved in PBS-EDTA) was added for 2–3 min. at 37°C; cell suspension was then collected and cell number was determined in a Newbauer chamber.

*Adipogenic and inflammatory markers in RPAT-SVF cells*. Precursor cells were obtained from CTR and MSG RPAT pads as described above. Thereafter, total RNA was isolated from cells by the single-step acid guanidinium isothiocyanate-phenol-chloroform extraction method (Trizol; Invitrogen, Life Tech., Waltham, MA, USA; cat. # 15596-026) [19]. It was then proceeded to quantify cell mRNA expression levels of different markers, such as: (a) those of AT precursor cell commitment (PPAR-γ2 and Zfp423); (b) key pro-adipogenic factors (GR and MR); (c) pro-inflammatory mediators (TNF-α, IL-6 and PAI-1); and (d) immune cell recruitment (CD11b, F4/80 and MCP-1). One μg of total RNA was reverse transcribed using random primers (250 ng) and Superscript III Rnase HReverse Transcriptase (200 U/Hl; cat # 18989-093; Invitrogen, Life Tech). Two μL of reverse transcription mix was amplified with 10 μl of QuantiTect SYBR green PCR solution kit (cat. # 204143; Qiagen, Waltham, MA, USA), in the presence of 1 μl of each specific primer (0.5 μM final concentration), and revealed using a LightCycler Detection System (MJ Mini Opticon, Bio-Rad, Hercules, CA, USA). Specific primers used are shown in alphabetical order in Table 1, where β-actin (ACTB) was the local reference gene marker. PCR efficiency was near 1. Threshold cycles (Ct) were measured in separate tubes, by duplicate. Identity and purity of the amplified product were checked by electrophoresis on agarose mini-gels and the melting curve was analysed at the end of amplification. The differences between Cts were calculated in every sample for each gene of interest as follows: Ct gene of interest−Ct ACTB gene. Relative changes in the expression level of a specific gene (ΔΔCt) were calculated as ΔCt of the test group minus ΔCt of the CTR group, then expressed as 2^−ΔΔCt^.

**Table 1:** Rat specific primers (in alphabetical order) for real-time PCR analyses (se: sense; as: anti-sense; GBAN: GenBank Accession Number; amplicon length, in bp)

		GBAN	bp
ACTB	se, 5′-AGCCATGTACGTAGCCATCC-3′	NM_031144	115
	as, 5′-ACCCTCATAGATGGGCACAG-3′		
ADIPOQ	se, 5′-AATCCTGCCCAGTCATGAAG-3′	NM_144744	159
	as, 5′-TCTCCAGGAGTGCCATCTCT-3′		
CD11b	se, 5′-CATCACCGTGAGTTCCACAC-3′	NM_012711.1	174
	as, 5′-GAGAACTGGTTCTGGCTTGC-3′		
C/EBPα	se, 5′-CTGCGAGCACGAGACGTCTATAG-3′	NM_012524	159
	as, 5′-TCCCGGGTAGTCAAAGTCACC-3′		
F 4/80	se, 5′-CAGCTGTCTTCCCGACTTTC-3′	NM_001007557.1	156
	as, 5′-TAATCAAGATTCCGGCCTTG-3′		
GR	se, 5′-TGCCCAGCATGCCGCTATCG-3′	NW_047512	170
	as, 5′-GGGGTGAGCTGTGGTAATGCTGC-3′		
IL-6	se, 5′-CTGATTGTATGAACAGCGATG-3′	NM_012589.2	140
	as, 5′-GAACTCCAGAAGACCAGAGC-3′		
LEP	se, 5′-GAGACCTCCTCCATCTGCTG-3′	NM_013076	192
	as, 5′-CTCAGCATTCAGGGCTAAGG-3′		
MCP-1	se, 5′-TCCACATTCGGAGGCTAAAG-3′	NM_001105822.1	183
	as, 5′-ACGTGAAGGTTCAAGGATGC-3′		
MR	se, 5′-TCGCTCCGACCAAGGAGCCA-3′	NM_013131	193
	as, 5′- TTCGCTGCCAGGCGGTTGAG-3′		
PAI-1	se, 5′-TGCCCCTCTCCGCCATCACC-3′	NM_012620.1	141
	as, 5′-TCTCCAGGGGCCCTCTGAGGT-3′		
PPAR-γ2	se, 5′-AGGGGCCTGGACCTCTGCTG-3′	NW_047696	185
	as, 5′-TCCGAAGTTGGTGGGCCAGA-3′		
Pref-1	se, 5′-TGCTCCTGCTGGCTTTCGGC-3′	NM_053744	113
	as, 5′-CCAGCCAGGCTCACACCTGC-3′		
TNFα	se, 5′-CATTCCTGCTCGTGGCGGGG-3′	NM_012675.3	177
	as, 5′-CGACGTGGGCTACGGGCTTG-3′		
Wnt-10b	se, 5′-AGGGGCTGCACATCGCCGTTC-3′	NW_047784	175
	as, 5′-ACTGCGTGCATGACACCAGCAG-3′		
Zfp423	se, 5′-CCGCGATCGGTGAAAGTTG-3′	NM_053583.2	121
	as, 5′-CACGGCTGGATTTCCGATCA-3′		

*RPAT-SVF cell differentiation*. As previously reported [19], a similar number of confluent (on Pd 5-6) SVF cells (100,000 cells, approximately) was induced to differentiate (Differentiation day zero; Dd 0) by adding 5 μg/ml insulin, 0.25 μM DXM and 0.5 mM 3-isobutyl-l-methylxanthine (IBMX) in DMEM-HEPES supplemented with 10% FBS and antibiotics. After 48 hrs, media were removed and replaced by fresh media containing insulin (5 μg/ml), 10% FBS and antibiotics. Cells were then kept in culture up to Dd 10 and media were replaced by fresh media every 48 hrs. Media were stored at −20°C until measurement of leptin (LEP) concentrations. Cell samples were harvested and processed on different Dds for complementary determinations (see below).

#### Adipogenesis-related markers in precursor cells undergoing differentiation

*Intracellular cell lipid staining*. Cells from Dd 0 throughout Dd 10 were washed with PBS and fixed with 10% formalin (for 10–15 min.) in PBS. Then, cells were quickly washed with isopropanol 60% and stained for 1 hr with Oil Red O solution (2:3 vol/vol H_2_O:isopropanol, containing 0.5% Oil Red O) [20]. After staining, cells were washed (×3) with PBS, and the dye from stained material was extracted by adding 200 μl of isopropanol (10 min.). To quantify cell lipid content, optical density (OD) of samples was determined at 510 nm in a spectrophotometer. Remaining cells were digested with 200 μl of 0.25% trypsin solution in PBS-EDTA (37°C, 24 hrs). Thereafter, cell suspension was centrifuged at 8000 × g (15 sec.) and the OD of supernatants was read at 260 nm for DNA quantification. Oil Red O absorbance values were standardized to reflect culture DNA.

*Cell adipokine release*. Media LEP concentrations (Dd 0-Dd 10) were determined by a previously described radioimmunoassay (RIA) [8]. The standard curve ranged between 50 and 12,500 pg/ml, and CVs intra- and inter-assay were 4–6% and 5–8% respectively.

*Cell adipogenesis gene-markers*. Total RNA was isolated from cells (harvested between Dd0 and Dd10) as described above. Thereafter, mRNA expression levels of several markers of adipogenesis were determined by quantitative PCR as described before. For this aim, the following factors were measured: adiponectin (ADIPOQ), C/EBP-α, LEP and PPAR-γ2; ACTB was used as a local reporter gene (details on these specific primers are shown in Table 1).

*Analysis of mature adipocytes*. Separately, a similar number of confluent RPAT-SVF cells from different groups (on Pd 5-6) were cultured (300,000 cells/well, approximately) on sterile cover glasses (in 6-well plates) and induced to differentiate as described above. On Dd 10, cells were fixed with 10% formalin solution for 1 hr at room temperature, and then stained with haematoxylin–eosin. The percentage of differentiated cells was calculated by counting the total number of cells and the number of cells containing lipid droplets, when visualized in a light microscope (after counting 200–250 total cells per cover glass, 40× magnification). Lipid-containing cells were assigned to 3-graded stages of maturation depending on the nucleus position [21], as: stage I (central), stage II (between centre and periphery) and stage III (fully peripheral). The percentage of cells corresponding to each stage of maturation was expressed in relation to the total number of differentiated cells. Image analysis was assessed using a light microscope and image analysis software (Image ProPlus 5.0, Rockville, MD, USA).

#### Experiment 2

Three weeks prior experimentation (day 39 of age), CTR and MSG animals (*n* = 10/12 rats per group) were submitted (under light ketamine anaesthesia and by the dorsal approach) to either bilateral adrenalectomy (ADX) or sham-operation (SHX). At the time of surgery, while MSG-ADX rats were sc implanted with 75 mg corticosterone pellets (MSG-ADX+Cort), CTR-SHX and MSG-SHX animals were sc implanted with placebo (P) pellets (CTR-SHX+P and MSG-SHX+P; Innovative Research of America, Toledo, OH, USA) [14]. We earlier established, as reported by the manufacturer, that ADX+Cort animals display a constant peripheral level (5–6 μg/dl) of Cort [14]. Animals were then returned to their home cages and MSG-ADX+Cort rats drank NaCl 0.9% solution until experimentation. Following sacrifice, RPAT pads were aseptically dissected and processed as described in Experiment 1.

### Peripheral metabolite measurements

Circulating levels of LEP, insulin and Cort were determined by specific RIAs as earlier described [8]. Circulating concentrations of aldosterone were measured using a commercial RIA kit (Diagnostic System Lab., Inc., Webster, TX, USA); the standard curve ranged between 20 and 1600 pg/ml, and intra- and inter-assay CVs were 4–8% and 8–11% respectively. Plasma levels of tumour necrosis factor-α (TNF-α) were determined by a commercial ELISA kit (Cat. No. 558535; BD Biosciences Pharmingen, San Diego, CA, USA; the standard curve ranged between 5 and 500 pg/ml).

### Statistical analysis

Data, expressed as mean values ± SEM, were analysed by anova (one-way, neonatal treatment; or two-way, neonatal treatment and post-natal treatment/surgery) followed by either the Fisher's test or the nonparametric Mann–Whitney test [19]. Morphometric data were analysed by the least significant difference test [8]. *P* values lower than 0.05 were considered statistically different.

## Results

### The intact MSG rat phenotype

As expected [11], MSG rats were hypophagic (recorded on the last experimental week and expressed as the 7-day average: 73.54 ± 4.41 kJ/day *versus* 93.41 ± 6.22 kJ/day in CTR rats; *P* < 0.05, *n* = 12–15 rats per group) and significantly (*P* < 0.05) lighter than CTR rats; however, RPAT pad mass was higher (*P* < 0.05 *versus* CTR) (Table 2). The peripheral concentrations of LEP and Cort were significantly (*P* < 0.05 *versus* CTR) higher in MSG animals (Table 2); conversely, no differences were noticed in those of insulin, TNF-α and aldosterone (Table 2). It is important to remark that MSG animals monitored at 30 days of age (*n* = 15–17 rats per group) had already developed hypophagia (5-day average: 40.44 ± 2.98 kJ/day *versus* 50.53 ± 3.32 kJ/day in CTR litter-mates; *P* < 0.05) and significantly (*P* < 0.05) higher plasma levels of Cort (18.17 ± 2.69 nM) and LEP (2.98 ± 0.23 ng/ml) after compared with CTR litter-mates (Cort: 6.79 ± 0.72 nM; and LEP: 1.08 ±0.11 ng/ml). These data clearly indicate that RPAT-SVF cells from MSG rats have been overexposed *in vivo* to Cort and LEP for a minimum period of 30 days.

**Table 2:** Bodyweight (BW), RPAT mass, peripheral levels of several metabolites (*n* = 12/15 rats per group) and AAT cell expression levels of pro-/anti-adipogenic genes (*n* = 4/5 different experiments, 4/5 replicates per experiment) in intact CTR and MSG male rats

	CTR	MSG
BW (g)	302.08 ± 9.41	235.41 ± 6.88*
RPAT Mass (g)	6.85 ± 0.71	13.07 ± 1.27*
RPAT Mass (g/100 g BW)	2.28 ± 0.31	5.53 ± 0.44*
Insulin (nM)	8.67 ± 0.94	11.56 ± 3.56
Leptin (ng/ml)	5.49 ± 0.79	15.23 ± 3.08*
TNF-α (pg/ml)	14.21 ± 5.42	19.87 ± 6.47
Corticosterone (nM)	7.79 ± 1.58	35.86 ± 7.01*
Aldosterone (pM)	95.25 ± 25.58	90.03 ± 21.97

Values are means ± SEM.

* *P* < 0.05 or less *versus* CTR values.

**Fig. 1:**
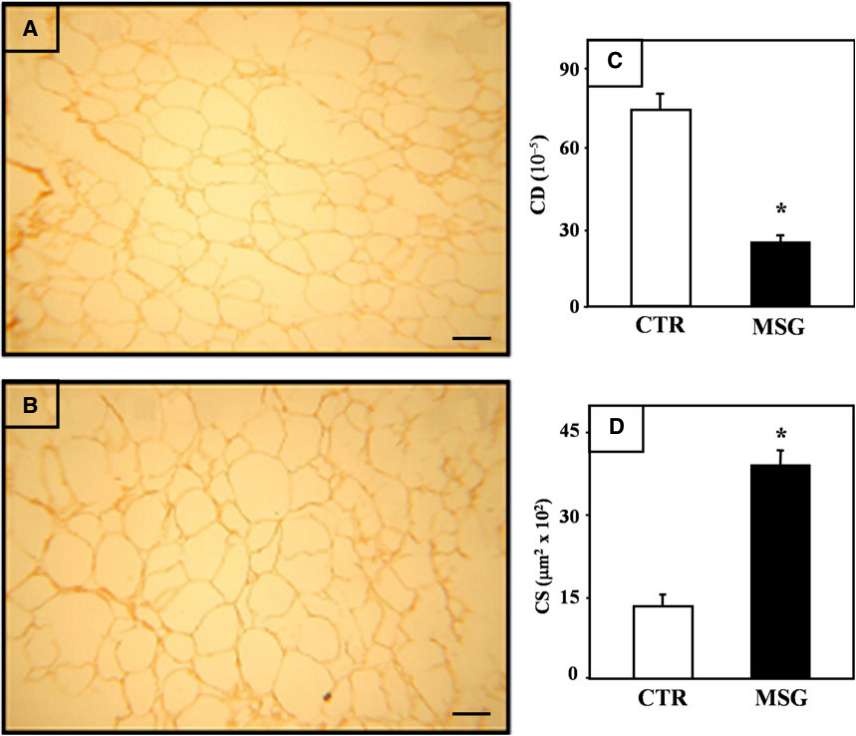
Representative fields of RPAT from intact CTR (**A**) and MSG (**B**) rats. Adipocytes, stained with haematoxylin–eosin, they all show cytoplasmic rim of mature adipocytes. Scale bar represents 50 μm. Morphometric parameters of RPAT adipocytes from intact CTR and MSG animals: cell density (CD) (**C**) and cell size (CS) (**D**). Values are the mean ± SEM (*n* = 4/5 rats per group). *P < 0.01 *versus* CTR values.

Figure 1 shows the histological characteristics of the RPAT pads (representative fields) from adult intact CTR (panel A) and MSG (panel B) male rats. Morphometric analyses indicate that MSG rats displayed a significant (*P* < 0.001 *versus* CTR) decrease in cell density (Fig. 1C) because of an increase in cell size (Fig. 1D).

### Precursor cell proliferation capacity

**Fig. 2:**
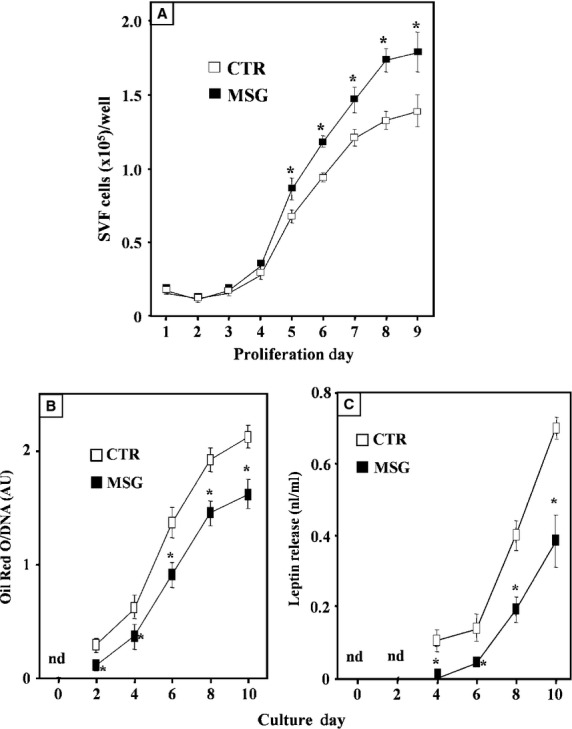
Proliferation curve of cultured RPAT-SVF cells, obtained from CTR and MSG rats (**A**). Lipid accumulation (**B**) and leptin secretion (**C**) by SFV cells, isolated from RPAT pads of adult CTR and MSG intact male rats, during *in vitro* differentiation. Values are means ± SEM (one animal per group per experiment; *n* = 3 different experiments, with 4/5 wells per day per experiment). *P < 0.05 *versus* CTR values.

After seed (Pd 0), the proliferation capacity of RPAT-SVF cells from either group was assessed by counting cell number every 24 hrs and plotting those numbers throughout the proliferation period (Pd 1–Pd 9). The results indicated (Fig. 2A) a significantly (*P* < 0.05) enhancement in the proliferation capacity of cells from the MSG group. In fact, this has already been noticed on Pd 5 and it lasted up to the end of the proliferation period (Pd 9). Moreover, the slopes of these curves were as follows: 18,877 ± 1457 and 25,670 ± 2055 cells per day, in CTR and MSG cell groups respectively (*P* < 0.05).

### Precursor cell differentiation capacity

Confluent RPAT-SVF cells (seed at 100,000 cells/well, approximately) from each group (CTR and MSG) were induced (on Dd 0) to *in vitro* differentiation and kept in culture up to Dd 10. Then, several functions and adipogenesis-related markers were studied in cells undergoing differentiation.

#### Lipidic and endocrine functions of cells undergoing differentiation

As intracellular lipid accumulation is a clear indicator of precursor cell differentiation, cell lipid content was evaluated every 48 hrs while differentiating. Although no cell lipid presence was noticed on Dd 0 in any group, the presence of lipidic vacuoles occurred in both cell groups on Dd 2 and later, an event corroborated after lipid quantification (Fig. 2B). Interestingly, cell lipid content was significantly (*P* < 0.05) lower in MSG than in CTR cells on all Dds. The spontaneous release of LEP by cells in culture was also evaluated, as a parameter of differentiation. As shown (Fig. 2C), medium LEP concentration was primarily detected on Dd 4 in both groups, although at a very low concentration in the MSG cell culture. Consistently, MSG cells released significantly (*P* < 0.05 *versus* CTR cells) lower amounts of LEP when analysed between Dds 6 and 10 (Fig. 2C).

#### Expression of key adipogenesis-related genes in cells undergoing differentiation

**Fig. 3:**
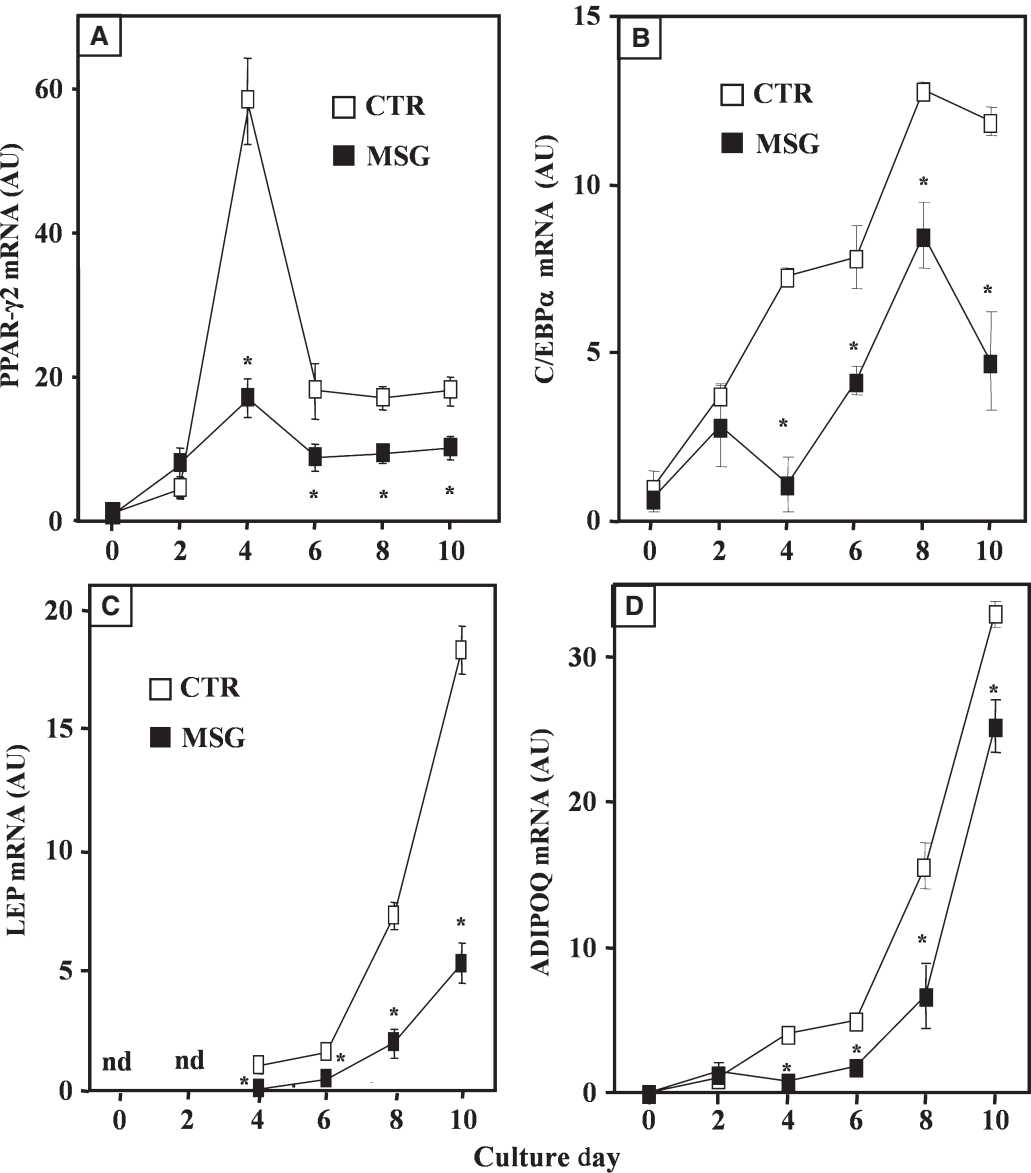
Cell mRNA expression levels (in arbitrary units: AU) of PPAR-γ2 (**A**), C/EBP-α (**B**), leptin (**C**) and ADIPOQ (**D**) during *in vitro* differentiation of isolated SVF cells isolated from RPAT pads of intact adult CTR and MSG male rats. Values are means ± SEM (one animal per group per experiment; *n* = 5/6 different experiments with 10/12 wells per day per experiment; nd: not detected). *P < 0.05 *versus* CTR values on the same day.

It is accepted that PPAR-γ2 is the main adipogenic factor driving the expression of other genes involved in the final step of adipogenesis. Although in minimal traces, cell PPAR-γ2 mRNA was detected on Dd 0 in both cell groups; cell PPAR-γ2 gene expression then increased rapidly (Dd 2) after inducing differentiation (Fig. 3A). This signal reached its highest expression level on Dd 4 in both groups; however, cell mRNA level was several fold lower (*P* < 0.05 *versus* CTR values) in MSG cells. Moreover, on Dd 6, expression of PPAR-γ2 gene had down-regulated in both cell groups, a pattern lasting up to Dd 10. Notably, PPAR-γ2 mRNA expression was significantly (*P* < 0.05) lower in MSG than in CTR cells throughout Dds 4–10 (Fig. 3A). The pattern of differentiating cell C/EBP-α mRNA level varied in a similar fashion to that of PPAR-γ2 gene expression, being its expression level significantly lower in MSG than in CTR cells (Fig. 3B). Accordingly to data from cell functionality, intracellular LEP mRNA appearance was first detected on Dd 4 in both groups; however, its expression level resulted significantly (*P* < 0.05) lower in MSG than in CTR cells throughout Dds 6–10 (Fig. 3C). Finally, ADIPOQ gene expression in differentiating cells was significantly lower in MSG (*versus* CTR) cells throughout Dds 4–10 (Fig. 3D).

### Morphometry of *in vitro* differentiated adipocytes

**Fig. 4:**
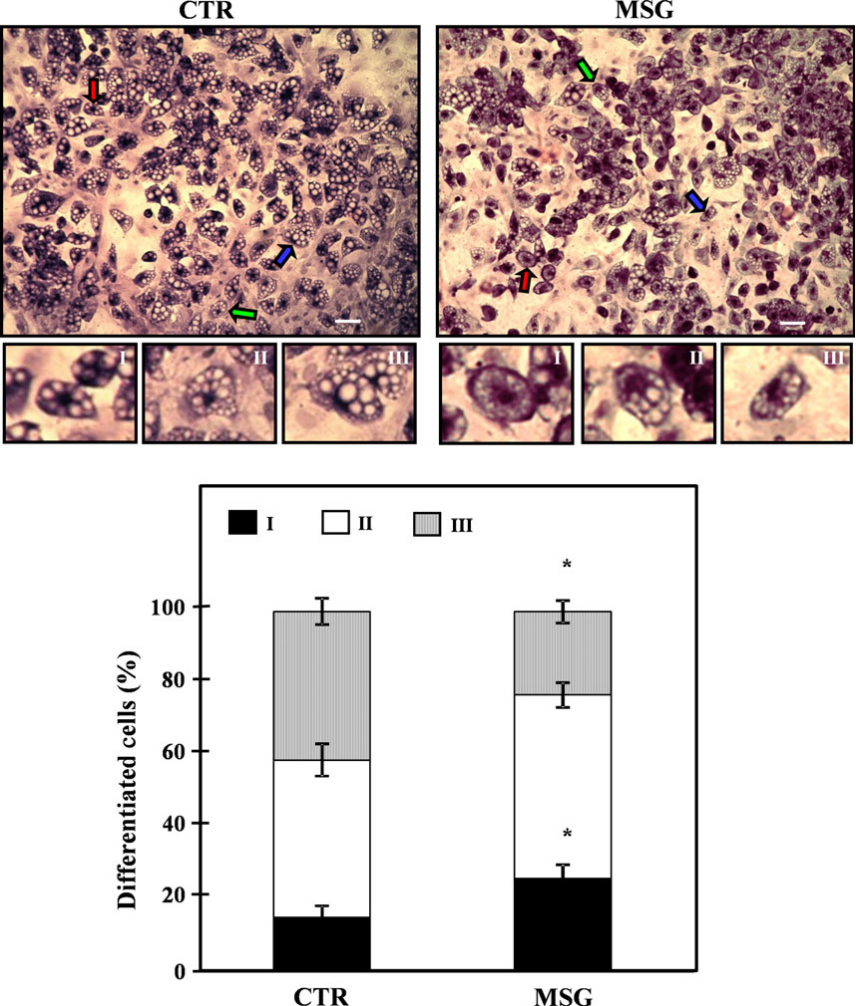
Representative fields containing *in vitro* differentiated CTR (upper left panels) and MSG (upper right panels) adipocytes (stained on Dd 10, magnification 10 × ), displaying different degrees of maturation depending on the nucleus position: I, central (white arrow); II, displaced from the centre (grey arrow); and III: fully peripheral (black arrow). The areas marked with arrows were enhanced to clearly show differences in the nucleus position (white bars: 50 μm). Percentages of *in vitro* differentiated (on Dd 10) CTR and MSG adipocytes according to their stages of maturation are also shown (lower panel). Values are means ± SEM (one animal per group per experiment; *n* = 4/5 different experiments; data from 200/250 cells were recorded in each experiment). *P < 0.05 *versus* CTR values.

Fixed cells on cover slips from Dd 10 were used to assess the percentage of cell differentiation and the *in vitro* degree of differentiation in both groups. Data indicate that in MSG cells, a significantly lower percentage of differentiated (lipidic vacuoles) cells was found: 62.9 ± 2.6% and 55.6 ± 2.9% in CTR and MSG groups respectively (*P* < 0.05; *n* = 4/5 different experiments; counting 40–50 cells per field from five different fields per experiment, thus summing 200–250 cells counted per experiment). In addition, the degree of maturation attained by differentiated cells according to their nuclear position as a parameter (Fig. 4, upper panels) indicated that MSG cells reached impaired cell maturation. Indeed, significantly (*P* < 0.05 *versus* CTR cells) higher and lower percentages of MSG cells in maturation stages I and III were detected respectively (Fig. 4, lower panel).

### Adipogenic and inflammatory status of RPAT precursor cells

**Fig. 5:**
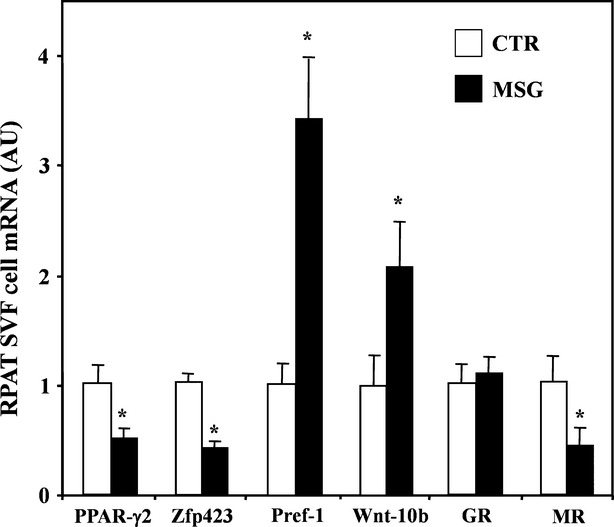
RPAT-SVF cell mRNA concentrations of adipogenesis commitment (PPAR-γ2 and Zfp423), anti-adipogenic (Pref-1 and Wnt.10b) and pro-adipogenic (GR and MR) markers. Precursor cells were isolated from RPAT pads of adult male, CTR and MSG rats. Values are means ± SEM (*n* = 5/6 specimens per group). *P < 0.05 *versus* CTR values.

Given our data supporting a clear and significantly impaired differentiation process of MSG RPAT-SVF cells, several adipogenesis-related parameters in freshly isolated precursor (SVF) cells from both groups were then evaluated. Data showing that the expression level of key markers of precursor cell commitment (PPAR-γ2 and Zfp423) was significantly (*P* < 0.05) lower in MSG- than in CTR-SVF cells are depicted in Figure 5. The latter Figure also shows that the mRNA levels of two anti-adipogenic transcription factors, Pref-1 and Wnt-10b, were three- and twofold higher, respectively, in MSG than in CTR cells (*P* < 0.05). Regarding the pro-adipogenic signals measured, although there was similar GR gene abundance in both cell groups, the MR mRNA expression level was significantly (*P* < 0.05 *versus* CTR) lower in MSG cells (Fig. 5).

As the expansion of the AT is often associated with an increased inflammatory state, various RPAT-SVF cell markers of inflammation and immune cell recruitment were evaluated. The results indicate that CTR and MSG precursor cells express similar mRNA levels of pro-inflammatory markers (PAI-1, TNF-α and IL-6) (Table 3). Similarly, no differences were noticed in the SVF cell expression levels of immune cell markers (MCP-1, F4/80 and CD11b) among cell groups (Table 3).

**Table 3:** Pro-inflammatory and immune cell markers mRNA levels in SFV cells from CTR and MSG animals

	SVF cell mRNA (AU)	
	
Marker	CTR	MSG
PAI-1	0.997 ± 0.116	0.745 ± 0.115
TNF-α	1.021 ± 0.237	0.627 ± 0.077
IL-6	1.011 ± 0.221	0.989 ± 0.159
MCP-1	0.989 ± 0.305	0.981 ± 0.127
F 4/80	0.976 ± 0.265	0.615 ± 0.092
CD11b	1.032 ± 0.341	1.139 ± 0.252

Values are means ± SEM (*n* = 4/5 samples per group; AU: Arbitrary Units).

### Impact of endogenous GC normalization in the MSG rat

Because of the overall impaired *in vitro* adipogenic capacity of RPAT-SVF cells from the MSG rat, it was explored whether such a dysfunction could be related to their high plasma Cort levels or not (Experiment 2). For this purpose, experiments were conducted in normo-corticosteronaemic (CTR-SHX-P and MSG-ADX+Cort) and hypercorticosteronaemic (MSG-SHX+P) rats.

#### Rat phenotype

MSG-ADX+Cort rats displayed similar BWs to those of MSG-SHX+P animals (Table 4). Conversely, RPAT mass in the MSG-ADX+Cort group was significantly (*P* < 0.05) lower than in MSG-SHX+P rats, thus resulting in similar values observed in CTR-SHX+P animals (Table 4). Conversely to that observed in the circulating levels of hormones in intact rats (see Table 2), the plasma Cort and LEP concentrations in MSG-ADX+Cort and CTR-SHX+P rats were similar, being significantly (*P* < 0.05) lower than those found in MSG-SHX+P rats (Table 4).

**Table 4:** Bodyweight (BW), RPAT pad mass, basal circulating levels of metabolites (*n* = 10/12 rats per group) and RPAT cell expression levels of pro-/anti-adipogenic genes (*n* = 4/5 different experiments, 4/5 replicates per experiment) in sham-operated control animals sc implanted with placebo pellet (CTR-SHX+P) and MSG rats, either sham operated or ADX, and sc implanted with placebo (MSG-SHX+P) or Cort (MSG-ADX+Cort) pellet

	CTR-SHX+P	MSG-SHX+P	MSG-ADX+Cort
BW (g)	278.61 ± 13.93	211.79 ± 11.78*	216.57 ± 8.66*
RPAT Mass (g/100 g BW)	2.53 ± 0.18	5.62.04 ± 0.56*	3.67 ± 0.38†
Corticosterone (nM)	20.21 ± 4.09	41.86 ± 8.86*	19.45 ± 2.14†
LEP (ng/ml)	4.82 ± 0.61	13.28 ± 2.03*	3.88 ± 0.45†
RPAT-SVF Cell PPAR-γ2 (AU)	1.01 ± 0.11	0.58 ± 0.05*	0.88 ± 0.07†
RPAT-SVF Cell Zfp423 (AU)	0.98 ± 0.08	0.53 ± 0.12*	0.91 ± 0.14†
RPAT-SVF Cell Pref-1 (AU)	0.99 ± 0.09	2.81 ± 0.16*	1.37 ± 0.17†
RPAT-SVF Cell Wnt-10b (AU)	1.02 ± 0.15	2.51 ± 0.44*	1.62 ± 0.29
RPAT-SVF Cell MR (AU)	1.03 ± 0.19	0.55 ± 0.15*	1.12 ± 0.09†

Values are the mean ± SEM.

* *P* < 0.05 *versus* CTR-SHX+P values.

† *P* < 0.05 *versus* MSG-SHX+P values.

#### RPAT precursor cell characteristics

Similar to that accounted in intact MSG animals (see Fig. 5), the expression levels of precursor cell commitment (PPAR-γ2 and Zfp423) and pro-adipogenic (MR) genes were significantly (*P* < 0.05 *versus* CTR-SHX+P) lower in the MSG-SHX+P group; moreover, those of anti-adipogenic signals (Pref-1 and Wnt-10b mRNAs) also were higher in MSG-SHX+P cells (*P* < 0.05 *versus* CTR-SHX+P; Table 4). Notably, PPAR-γ2 and Zfp423 mRNAs concentrations in precursor cells were fully corrected by normalizing the circulating levels of GC in MSG rats (MSG-ADX+Cort; Table 4). The enhanced precursor cell expression levels of anti-adipogeneic genes (Pref-1 and Wnt-10b) were fully and partly, respectively (*P* < 0.05), prevented by corticosterone-replacement therapy in ADX MSG rats (Table 4). Interestingly, RPAT-SVF cells from MSG-ADX+Cort displayed a complete (*P* < 0.05 *versus* MSG-SHX+P) restoration of MR mRNA expression up to values found in CTR-SHX+P cells (Table 4), while cell GR gene abundance remained unchanged (data not shown).

#### RPAT precursor cell proliferative capacity

Cort-replacement therapy in ADX MSG (MSG-ADX+Cort) rats resulted in a significant (*P* < 0.05) decrease in SVF cell proliferation (*n* = 3/4 experiments), as analysed on Pd 5 (95,142 ± 2320 cells) and compared with MSG-SHX+P cells (118,119 ± 4791 cells). Moreover, the results were similar to that displayed by CTR-SHX+P cells (94,114 ± 3009 cells).

#### RPAT precursor cell adipogenic capacity

**Fig. 6:**
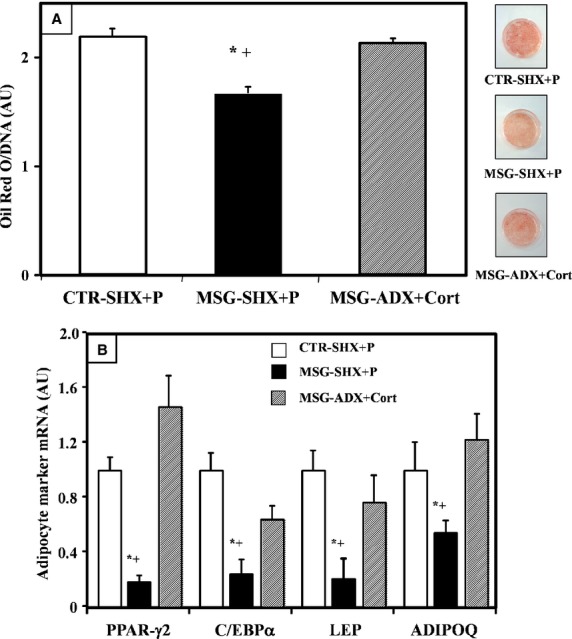
Cell lipid content (**A**) and adipogenesis-related gene expression (**B**) in different groups (CTR-SHX+P, MSG-SHX+P and MSG-ADX+Cort rats) evaluated on differentiation 10. Values are means ± SEM (one animal per group per experiment; *n* = 4/5 different experiments, with 5 wells per experiment). *P < 0.05 *versus* CTR-SHX+P values; +*P* < 0.05 *versus* MSG-ADX+Cort values.

**Fig. 7:**
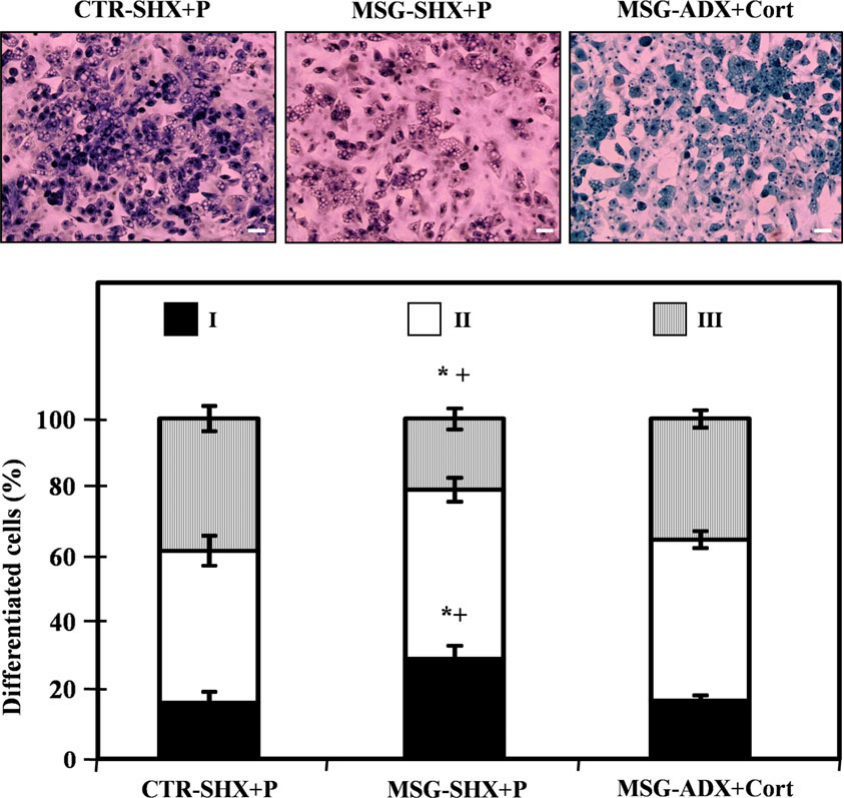
Representative fields containing *in vitro* differentiated adipocytes from different groups (CTR-SHX+P, MSG-SHX+P and MSG-ADX+Cort rats; upper panels). Cells were stained on Dd 10, magnification 10 × and display different degrees of maturation depending on the nucleus position (for more details, see legend in Fig. 4): I, central; II, displaced from the centre; and III: fully peripheral (white bars: 50 μm). Percentages of *in vitro* differentiated (on Dd 10) CTR-SHX+P, MSG-SHX+P and MSG-ADX+Cort adipocytes according to their stages of maturation are also shown (lower panel). Values are means ± SEM (one animal per group per experiment; *n* = 4/5 different experiments; data from 200/250 cells were recorded in each experiment). *P < 0.05 *versus* CTR-SHX+P values; +*P* < 0.05 *versus* MSG-ADX+Cort values.

When measured on Dd 10, lipid content was significantly (*P* < 0.05) higher in MSG-ADX+Cort than in MSG-SHX+P cells, and it was similar to that of CTR-SHX+P cells (Fig. 6A). On Dd 10, the reduced (*P* < 0.05 *versus* CTR-SHX+P cells) expression of key marker genes (PPR-γ2, C/EBP-α, LEP and ADIPOQ) in MSG-SHX+P cells was fully corrected after normalization of plasma Cort levels in MSG rats (MSG-ADX+Cort; Fig. 6B). The analysis of the percentages of mature adipocytes (Dd 10) indicated a significantly (*P* < 0.05) lower percentage in the MSG-SHX+P (57.8 ± 3.1%) than in the CTR-SHX+P (65.5 ± 2.7%) group, a parameter similar to that recorded in MSG-ADX+Cort cells (60.9 ± 2.1%). Finally, the analysis of the degree of maturity reached on Dd 10 in MSG-ADX+Cort cells was characterized by lower and higher percentages of differentiated cells in stages I and III, respectively, than those recorded in the MSG-SHX+P cell group (Fig. 7). Interestingly, the pattern found in MSG-ADX+Cort cells was similar to that displayed by the CTR-SHX+P cell group (Fig. 7).

## Discussion

Our study indicates that SVF cells isolated from the RPAT of the hypertrophic obese, MSG rat possess an impaired capacity to differentiate *in vitro*. This dysfunction seems to be mainly related to their *in vivo* chronic overexposure to a GC-rich environment. The adipogenic process in 60-day-old MSG rats was studied because, when 90 days of age was reached, there was a deteriorated peripheral pattern of other adipogenesis-related signals [8,14,17] and development of morbid adiposity, a state characterized by deeply dysfunctional enlarged adipocytes that release high amounts of leptin [8], with impaired insulin sensitivity [8] and an enhanced lipogenic activity [16]. However, we have presently addressed that hypertrophic AT mass expansion also takes place at an earlier age.

Zfp423 is a signal that regulates AT precursor cell commitment and, in turn, PPAR-γ2 expression [1]; moreover, Zfp423 acts as a co-activator of Smad proteins and amplifies the pro-adipogenic and PPAR-γ2-inducing activities [22]. In this regard, our data indicate that RPAT precursors isolated from MSG rats under-expressed Zpf423 and PPAR-γ2 genes, thus suggesting a lower abundance of AT-committed cells [1] in their RPAT SVF. The MSG phenotype carries high peripheral Cort levels and their RPAT-SVF cells display both enhanced proliferation capacity and reduced MR mRNA levels. Given that GCs are inhibitors of cell proliferation [23], these data could suggest that a state of GC resistance has been established at the precursor cell level. Nevertheless, a high leptinaemia-dependent effect on SVF cell proliferation should not be discarded [24]. Indeed, the diminished PPAR-γ2 gene expression in differentiating MSG cells could partly be related to their high leptinaemia. In fact, it has been shown that AT precursor cells from *db/db* mice, transgenic for *ob*-Rb [25], overexpress Pref-1 mRNA, as occurred in MSG rats; interestingly, those mice display retarded adipogenesis [25]. As previously described [26], the adrenal leptin resistance in MSG rats can be overridden by bilateral ADX-reduced leptinaemia. Therefore, hyperleptinaemia could be another direct/indirect factor regulating MSG RPAT-SVF cell expression of Pref-1/Wnt [25] and thus adipogenesis [27].

A bi-directional link between obesity and inflammation is now accepted [28,29]. As known, AT inflammation contributes to the development of several deleterious metabolic effects in obesity and later cooperates with the development of insulin resistance [30]. TNF-α and IL-6, two well-known pro-inflammatory cytokines, affect insulin action and promote insulin resistance [31–33]. In turn, this local (AT) pro-inflammatory environment induces the recruitment of immune cells. However, the expression of several cell markers of inflammation and immune cell recruitment in RPAT-SVF cells remain unaltered in MSG rats; moreover, similar results in RPAT pads from these rats were also found (data not shown). It is accepted that the activation of GR receptors in AT inhibits the pro-inflammatory response, while MR activation induces an anti-inflammatory activity [34,35]. It is highly probable that the altered balance between GR and MR in MSG RPAT-SVF cells could be, at least partially, responsible for the lack of an enhanced pro-inflammatory activity in their hypertrophic RPAT pads. There are published data tallying with our findings with reference to a lack of an installed inflammatory state in different obese phenotypes. As example, it has been described that DXM treatment in high-fat diet-induced obese mice inhibits their immune response [36] and that the adult MSG male rat did secrete less TNF-α than normal rats during acute endotoxaemia [17]. Moreover, human patients with Cushing's syndrome display increased adiposity but not any chronic inflammatory state [37,38].

As seen in other obese phenotypes [39,40], the impaired differentiation capacity of MSG RPAT-SVF cells could result from changes in pre-adipocyte sensitivity to inducers of differentiation [39]. Indeed, DXM stimulation is a crucial signal activating precursor cell PPAR-γ2 mRNA expression [41], a key cell-marker triggering final steps of the differentiation process. It was found, on the normal peak day (Dd 4 in CTR cells) of cell PPAR-γ2 gene abundance, that this cell marker was very low in the MSG cell population. Interestingly, through differentiation of MSG precursor cells, their metabolic (lipidic) functionality first appeared, as in normal cells, on Dd 2 and coincidentally with PPAR-γ2 expression. However, MSG cells resulted metabolically dysfunctional, and later (Dd 4), the malfunction accounted for their endocrine function (leptin gene abundance/leptin secretion). These dysfunctions could be indicative for a disrupted final sequence of events up to reach the mature adipocyte phenotype. Nevertheless, the departing reduced MSG RPAT precursor cell MR expression seems to be the first crucial step affected throughout adipogenesis.

Our data that indicate a clear relationship between low MSG RPAT-SVF cell MR expression and impaired cell differentiation fully agree with previous reports. Indeed, when confluent MR-expressing 3T3-L1 cells were induced to differentiate *in vitro* and then cultured with aldosterone alone, cells enhanced their PPAR-γ2 mRNA expression, accumulated lipid and produced adipokines [2]. Interestingly, the authors showed that these events were fully prevented by co-incubating cells with spironolactone or by knocking-down cell MR [2], but not by silencing GR [2]. In line with these observations, a mouse pre-adipocyte cell line lacking MR overexpresses Pref-1, despite no changes in Pref-1 when these cells were knockout for GR [35]. However, an appropriate cell balance in MR/GRs determines both the phenotype of mature adipocytes [35] and the regional distribution of them [42]. All together, this evidence highlights a pivotal role of MR in corticoid-induced adipogenesis [2,35,42,43].

It was previously observed that correcting GC excess in MSG rats [8,14], a full reversion of several metabolic and endocrine dysfunctions accounted, thus clearly indicating that GC overproduction plays a key role not only in the development but also in the maintenance of the MSG rat phenotype. GC is a potent adipogenic factor [3,4] because of its inhibitory activity on precursor cell Pref-1 [44]. AT precursor cell differentiation requires the suppression/repression of Pref-1/Wnt-10b [3,4]. Although a clear suppressive effect of GC on precursor cell Pref-1 is accepted [3,4], conversely, little is known regarding any GC regulatory activity on Wnt functionality, namely at earlier steps of adipogenesis. In this respect, MR/GRs interact with elements of the Wnt signalling pathway [45], and its activation favours precursor cell differentiation [46]. However, it is also accepted that enhanced Wnt-10b signalling inhibits adipogenesis [47]. During cell trans-differentiation, a transient suppression of Wnt gene expression/signalling is required, and it has been established that an excess of GC is able to inhibit this signal [48]. Reduced MSG RPAT-SVF cell MR expression could be responsible for a low corticoid inhibitory effect on Pref-1/Wnt-10b, thus resulting in an overall impaired adipogenic process. We also addressed that this cell dysfunction is, at least partly, dependent on a rich corticosterone milieu. Indeed, our experiments with RPAT-SVF cells from MSG rats devoid of chronic high peripheral GC levels indicated that cells recovered normal proliferation and Zfp423/PPAR-γ2 gene expression and balanced mRNA expression levels of anti-(Pref-1/Wnt-10b) and pro-(MR) adipogenic signals. Moreover, fully differentiated MSG-ADX+Cort adipocytes accumulated lipid, expressed key adipogenic-marker genes and displayed a degree of maturity similar to that occurred in CTR (normal) cells.

In conclusion, an impaired adipogenic process taking place in the adult MSG rat was addressed, a dysfunction dependent on the endogenous GC-rich environment. Such an impaired process could be inducing to an unhealthy [18,49], hypertrophic AT mass expansion. The latter could be triggered by precursor cells overexpressing Pref-1, as found in MSG rats and other human obese phenotypes [50]. The study further supports a MSG RPAT-SVF cell mis-programming, a failure related to a GC-dependent basis rather than to any epigenetic modification. This highlights the clinical relevance of correcting corticoadrenal hyperactivity to counteract AT-induced metabolic-endocrine dysfunctions in Cushing's syndrome and other hypertrophic obese phenotypes [10,18,49,50].
